# Efficacy and Tolerability of Vortioxetine Versus Selective Serotonin Reuptake Inhibitors for Late-Life Depression: A Post-hoc Analysis of the VESPA Study

**DOI:** 10.1007/s40266-025-01231-3

**Published:** 2025-07-18

**Authors:** Francesco Bartoli, Daniele Cavaleri, Ilaria Riboldi, Tommaso Callovini, Cristina Crocamo, Chiara Gastaldon, Andrea Aguglia, Camilla Callegari, Simone Cavallotti, Stefania Chiappini, Marco Cruciata, Armando D’Agostino, Irene Espa, Luigi Grassi, Marta Ielmini, Silvia Mammarella, Giovanni Martinotti, Marianna Rania, Alessandro Rodolico, Rita Roncone, Valentina Roselli, Cristina Segura-Garcia, Maria Salvina Signorelli, Lorenzo Tarsitani, The VESPA Study Group, Giovanni Ostuzzi, Giuseppe Carrà

**Affiliations:** 1 School of Medicine and Surgery, University of Milano-Bicocca, via Cadore 48, 20900 Monza, Italy; 2 Section of Psychiatry, Department of Neuroscience, Biomedicine and Movement Sciences, World Health Organization Collaborating Centre for Research and Training in Mental Health and Service Evaluation, University of Verona, Verona, Italy; 3 Section of Psychiatry, Department of Neuroscience, Rehabilitation, Ophthalmology, Genetics, Maternal and Child Health, University of Genoa, Genoa, Italy; 4 IRCCS Ospedale Policlinico San Martino, Genoa, Italy; 5 Section of Psychiatry, Department of Medicine and Surgery, University of Insubria, Varese, Italy; 6 Department of Mental Health and Addiction, ASST Santi Paolo e Carlo, Milan, Italy; 7 Department of Neurosciences, Imaging and Clinical Sciences, University “G. D’Annunzio”, Chieti, Italy; 8 UniCamillus International Medical Sciences University, Rome, Italy; 9 Department of Neuroscience and Rehabilitation, Institute of Psychiatry, University of Ferrara, Ferrara, Italy; 10 Department of Health Sciences, University of Milan, Milan, Italy; 11 Department of Life, Health and Environmental Sciences, University of L’Aquila, L’Aquila, Italy; 12 Outpatient Unit for Clinical Research and Treatment of Eating Disorders, University Hospital Renato Dulbecco, Catanzaro, Italy; 13 Department of Clinical and Experimental Medicine, Institute of Psychiatry, University of Catania, Catania, Italy; 14 Department of Psychiatry and Psychotherapy, Technical University of Munich, TUM School of Medicine and Health, Klinikum rechts der Isar, Munich, Germany; 15 Department of Human Neuroscience, Sapienza University of Rome, Rome, Italy; 16 Psychiatry Unit, Department of Medical and Surgical Sciences, University Magna Graecia of Catanzaro, Catanzaro, Italy; 17 Oasi Research Institute-IRCCS, Troina, Italy

## Abstract

**Background and Objectives:**

Usual treatment approaches for late-life depression primarily involve selective serotonin reuptake inhibitors (SSRIs). Recently, the potential role of vortioxetine has garnered attention. This study aimed to investigate whether vortioxetine is superior to SSRIs in terms of efficacy and tolerability in older people with moderate-to-severe depression.

**Methods:**

The Vortioxetine in the Elderly versus SSRIs: a Pragmatic Assessment (VESPA) study was an assessor-blinded, randomized, parallel-group, superiority trial, comparing flexible doses of vortioxetine versus SSRIs in older adults with depression. This is a post-hoc analysis that excluded participants with milder symptoms of depression. The primary outcome was the change in Montgomery–Åsberg Depression Rating Scale (MADRS) scores. Secondary outcomes included clinical response (MADRS total score reduction of ≥ 50%), remission (a MADRS score < 10), and discontinuation rates. Clinical measures were conducted at baseline and at 1-month, 3-month, and 6-month (endpoint) visits.

**Results:**

In total, 302 individuals (mean age: 73.4 ± 5.9 years; 68.9% females), comprising 152 randomized to vortioxetine and 150 to SSRIs (sertraline *N* = 92; paroxetine *N* = 19; escitalopram *N* = 19; citalopram *N* = 16; fluoxetine *N* = 3; fluvoxamine *N* = 1), were included in this post-hoc analysis. No significant differences in MADRS improvement between vortioxetine and SSRIs were observed at any follow-up visits and 6-month endpoint (−11.8 ± 10.6 versus −14.0 ± 11.6; *p* = 0.12). This was further confirmed by a subgroup analysis excluding drug discontinuers (−16.8 ± 9.0 versus −17.6 ± 10.3; *p* = 0.51). In addition, people treated with vortioxetine did not exhibit better rates of response (44.1 versus 53.0%; *p* = 0.11), remission (25.7 versus 34.7%; *p* = 0.09), and discontinuation (38.0 versus 30.2%; *p* = 0.17), including discontinuation owing to either side effects or inefficacy, compared with those treated with SSRIs.

**Conclusions:**

Vortioxetine was not superior to SSRIs in terms of efficacy and tolerability in older adults with moderate-to-severe depression. Additional trials, possibly based on fixed doses of vortioxetine, are needed.

**Registration:**

Clinicaltrials.gov: NCT03779789, registered on 12 Dec 2018; EudraCT number: 2018-001444-66.

**Supplementary Information:**

The online version contains supplementary material available at 10.1007/s40266-025-01231-3.

## Key Points

**Table:** 

In this post-hoc analysis we found that vortioxetine did not exhibit better symptom improvement than SSRIs in old people with major depressive disorder.
Findings showed no superior response, remission, and discontinuation rates of vortioxetine over SSRIs.
Future trials, possibly using fixed and targeted doses, should clarify the role of vortioxetine, compared to SSRIs, in the management of late-life depression.

## Introduction

Major depressive disorder occurs in over 10% of older adults [1] and is generally associated with poor outcomes [2]. Treatment approaches for late-life depression are routinely based on selective serotonin reuptake inhibitors (SSRIs), generally representing the first-line pharmacological option [3]. However, a role for vortioxetine in the treatment of late-life depression has also recently been proposed [4]. Vortioxetine has been approved by the Food Drug Administration (FDA) and the European Medicines Agency (EMA) for the treatment of major depressive disorder in adults with a recommended dose range of 5–20 mg/day [5, 6]. Its clinical action is mediated mainly by selective blockade of serotonin reuptake (by inhibiting the serotonin transporter) and direct modulation of 5-hydroxytryptamine (5-HT) receptors activity (such as 5-HT_3_, 5-HT_7_, 5-HT_1D_ and 5-HT_1B_) [7]. Providing at least partial support for the hypothesis that vortioxetine may have a more favourable tolerability profile [8], post-hoc analyses of randomized trials have shown that it might be efficacious and well-tolerated in people aged ≥ 55 with major depressive disorder [9]. More recently, a small, double-blind, randomized trial ascertained comparable efficacy and tolerability between vortioxetine and sertraline in 50 older adults with major depressive disorder [10]. Conversely, the large pragmatic, assessor-blinded, randomized, multicentre Vortioxetine in the Elderly versus SSRIs: a Pragmatic Assessment (VESPA) study found that, contrary to the primary hypothesis, vortioxetine did not demonstrate a better tolerability profile than SSRIs in older adults with major depressive disorder [11]. Moreover, secondary efficacy outcomes revealed no significant differences between treatment arms in Montgomery–Åsberg Depression Rating Scale (MADRS) [11]. However, this primary study included also subjects with milder symptoms of depression with a MADRS baseline score below the standard cut-off of 20 [12], potentially limiting the generalizability of findings to people with moderate-to-severe depression. Baseline symptom severity might be a clinical predictor of antidepressant effects [e.g. 13, 14], and the use of antidepressant agents for treating milder variants of depression has been questioned [15]. Therefore, to explore whether vortioxetine is superior to SSRIs in individuals with more severe symptoms, we conducted an exploratory, post-hoc analysis of data from the VESPA study, aiming at investigating the efficacy and tolerability of vortioxetine versus SSRIs specifically focusing on older people with moderate-to-severe depression.

## Methods

### Study Design and Protocol

This exploratory, post-hoc study is a secondary analysis of the VESPA study data. The VESPA study is a pragmatic, assessor-blinded, randomized, parallel-group, superiority multicentre trial. It was funded by the Italian Medicines Agency (Agenzia Italiana del Farmaco – AIFA) in the context of the 2016 call for Independent Research on Drugs (code: 2016-0234923). The study was designed according to the principles of the CONsolidated Standards of Reporting Trials (CONSORT) statement [16]. Full details on study protocol, design, and procedures are reported elsewhere [11, 17]. The study was conducted between February 2019 and February 2023 in inpatient and outpatient Mental Health Services and was run by 11 national academic centres, i.e. University of Verona, University of Catania, Magna Graecia University of Catanzaro, University of Ferrara, University of Genova, University of Chieti-Pescara, Insubria University of Varese and Como, University of L’Aquila, Sapienza University of Rome, University of Milan, and University of Milano-Bicocca. The study was first approved for the coordinating centre (University of Verona) by the Ethics Committee for Clinical Research of Verona and Rovigo (protocol 61211 of the 19/09/2018; protocol version 1.5 of the 09/06/2018) and thereafter by the local ethics committee of each recruiting centre. The study protocol was registered in ClinicalTrials.gov (NCT03779789) on 12 December 2018 and published in advance [17].

### Inclusion Criteria

Participants were included in this study if a treatment with an antidepressant agent was considered clinically indicated. Individuals were eligible if they: (i) were aged 65 years or older, (ii) had a major depressive episode, according to the Diagnostic and Statistical Manual for Mental Disorders–fifth edition (DSM-5) criteria [18], and (iii) were willing to participate by signing an informed consent form. We excluded individuals with a formal diagnosis of dementia, bipolar or schizophrenia-spectrum disorders, as well as those treated with psychopharmacological agents that might influence depressive symptoms at the time of randomization, such as antidepressants, second-generation antipsychotics, and lithium. Moreover, for the specific purpose of the current study, individuals were included only if they had a baseline MADRS score ≥ 20 corresponding to the threshold for a moderate-to-severe depression [19, 20]. People with milder symptoms of depression were thus excluded.

### Procedures

Investigators consecutively enrolled participants from inpatient and outpatient services of each recruiting centre. Participants were randomly allocated to either vortioxetine or an SSRI chosen according to clinical judgment, with an allocation ratio of 1:1. A web-based application was used to manage the randomization process, employing a centralized procedure on the basis of a sequence of treatments randomly permuted in blocks of constant size (random even sizes between 2 and 8). This allocation sequence and the block size were concealed to study investigators. Allocation was stratified by the recruiting centre. For participants allocated to the SSRI group, the clinicians selected the SSRI (sertraline, citalopram, escitalopram, paroxetine, fluoxetine or fluvoxamine) that they deemed most appropriate according to each participant’s characteristics. A flexible dose of either vortioxetine or SSRI within the licensed dose range, as reported by AIFA, was allowed. The ratio between the prescribed daily dose (PDD) and the defined daily dose (DDD), according to the World Health Organization (WHO) ATC/DDD index [21], was used to estimate the equivalent daily doses of both vortioxetine and SSRIs. Drugs could be administered in tablets or drops. After randomization and throughout the trial, participants and clinicians were aware of treatment allocation, while outcome assessors operated under blinded conditions.

### Clinical Assessments and Outcomes

We collected clinical data and administered rating scales at baseline and at each follow-up visit, which took place at one (T1), three (T2), and six (T3) months after randomization. The MADRS was used to evaluate depressive symptoms at baseline and follow-up assessments. For the specific purpose of this post-hoc analysis, the primary outcome was the improvement of depressive symptoms as measured by change in MADRS score at follow-up assessments (T1, T2, and T3). Secondary outcomes at the study endpoint (T3) included; (i) clinical response (i.e. number of participants with a reduction in MADRS total score of ≥ 50%), (ii) clinical remission (i.e. number of participants with a MADRS score < 10) [22], (iii) any cause discontinuation (i.e. the number of participants discontinuing the assigned antidepressant), (iv) discontinuation due to side effects at T3, and (v) discontinuation due to inefficacy.

### Data Analysis

Analyses on the primary outcome (MADRS mean change score), as well as on clinical response and remission, were based on an intention-to-treat (ITT) approach, including all subjects regardless of drug discontinuation. A minimum of 141 participants per treatment arm was required for this post-hoc, exploratory analysis (alpha = 0.05; power = 80%), assuming a 3-point difference in MADRS mean change score between vortioxetine and SSRIs. To address missing data for participants with incomplete MADRS assessments or who discontinued the allocated treatment, the Last Observation Carried Forward (LOCF) method was employed. In addition, to more effectively handle missing data, a repeated-measures mixed-effects model analysis was performed, which accommodates unbalanced data under the assumption that data were missing at random.

In addition, subgroup analyses were carried out excluding individuals who either discontinued the drug or had missing information on discontinuation. For any cause and specific reason (due to side effects or inefficacy) discontinuation, a per-protocol (PP) analysis, excluding participants with missing information on this outcome, was carried out. Continuous variables were expressed as mean ± standard deviation (SD), while categorical variables as frequencies (%). Normality and heteroskedasticity assumptions for continuous data were assessed with Shapiro-Wilk and Levene’s tests, respectively. Continuous variables were compared using unpaired Student’s *t*-test or Mann–Whitney *U* test according to data distribution, while categorical variables were compared with chi-squared test or Fisher’s exact test. Moreover, survival analyses, estimating hazard ratio (HR) and related 95% confidence interval (CI), were used to test the differences in time of discontinuation between vortioxetine and SSRIs, using competing-risks regression to assess the occurrence of event discontinuation while taking into account death as a competing risk. Multiple regression analyses were used to test if PDD/DDD might influence endpoint outcomes (MADRS mean change score, response, remission, and discontinuation). The significance level was set at 5% and two-tailed tests were used. Statistical analyses were performed using Stata (StataCorp. 2023. Stata Statistical Software: Release 18. College Station, TX: StataCorp LLC).

## Results

### Study Participants

**Fig. 1:**
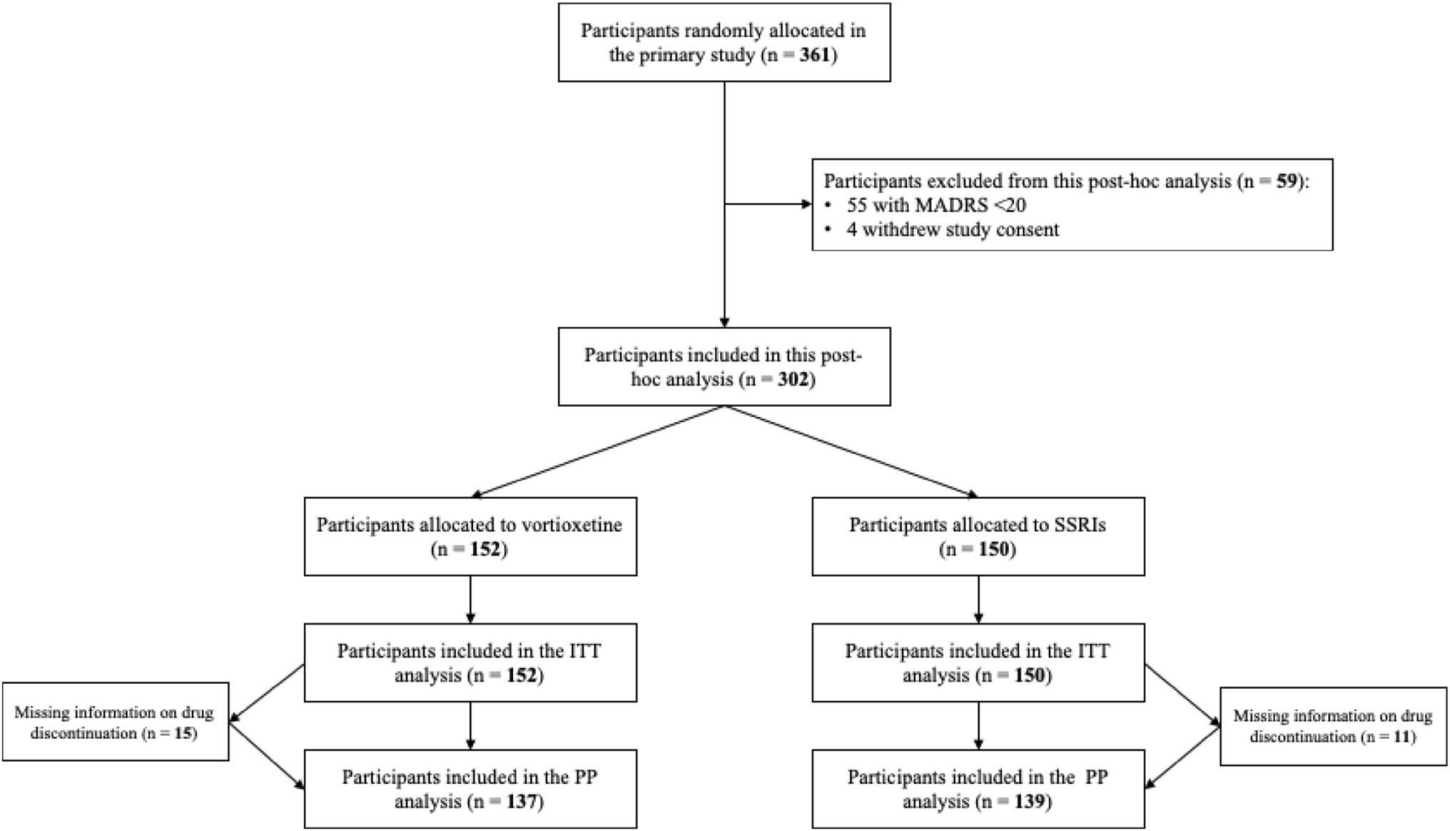
Flowchart of study participants. *n* number of study participants with the index variable, *MADRS* Montgomery–Åsberg Depression Rating Scale, *SSRIs* selective serotonin reuptake inhibitors, *ITT* intention-to-treat, *PP* per-protocol

A total of 361 individuals agreed to participate by signing the written informed consent and were included in the primary study [11]. For the specific purpose of this post-hoc analysis, 55 individuals (27 randomized to vortioxetine and 28 to a SSRI) were excluded since they had milder symptoms of depression (MADRS score < 20). In addition, four study participants (two randomized to vortioxetine and two to a SSRI) were excluded since they withdrew the study consent. Thus, 302 individuals, including 152 randomized to vortioxetine and 150 to SSRIs, were eligible for this post-hoc analysis and were included in the ITT sample. The PP sample was composed by 276 study participants, since no information on discontinuation rates was available from 26 individuals. The study participant flow diagram is reported in Fig. 1.

Study participants had a mean age of 73.4 ± 5.9 years and were mostly females (*N* = 208; 68.9%), with neither age nor sex differences between those treated with vortioxetine and those with SSRIs. Other sociodemographic characteristics were also comparable between groups. The baseline mean MADRS score was 29.8 ± 6.3 (29.7 ± 6.1 for participants allocated to vortioxetine and 29.8 ± 6.6 for those allocated to SSRIs). Among participants allocated to SSRI treatment, sertraline was the most prescribed SSRI (*N* = 92), while the others were treated with paroxetine (*N* = 19), escitalopram (*N* = 19), citalopram (*N* = 16), fluoxetine (*N* = 3), and fluvoxamine (*N* = 1). Participants randomized to vortioxetine (PDD/DDD = 0.84 ± 0.43) had lower (*p* < 0.001) PDDs than those randomized to SSRIs (PDD/DDD = 1.04 ± 0.28). Similar trends (*p* < 0.001) were confirmed over the course of the trial, in which the maximum prescribed PDD/DDD in the SSRI group (1.24 ± 0.48) was higher than that in the vortioxetine group (1.01 ± 0.45). In total, nine patients (five randomized to vortioxetine and four to a SSRI) died before the study endpoint. Full details on baseline characteristics of study participants are reported in Table 1.

**Table 1:** Characteristics of study participants

	Vortioxetine group (*n* = 152)	SSRI group (*n* = 150)
**Socio-demographic characteristics**		
Age, mean (SD), years	73.5 (6.1)	73.3 (5.7)
Male sex, *n* (%)	49 (32.2)	45 (30.0)
Education years, mean (SD)^a^	9.0 (4.1)	9.4 (4.2)
Married, *n* (%)^b^	74 (50.0)	83 (56.5)
**Physical comorbidities**		
Diabetes, *n* (%)	30 (19.7)	21 (14.0)
Myocardial infarction, *n* (%)	10 (6.6)	8 (5.3)
Congestive heart failure, *n* (%)	10 (6.6)	7 (4.7)
Stroke, *n* (%)	7 (4.6)	7 (4.7)
Peripheral vasculopathy, *n* (%)	33 (21.7)	30 (20.0)
Chronic obstructive pulmonary disease, *n* (%)	13 (8.5)	12 (8.0)
Liver disease, *n* (%)	8 (5.3)	11 (7.3)
Kidney disease, *n* (%)	3 (2.0)	6 (4.0)
Connective tissue disease, *n* (%)	19 (12.5)	20 (13.3)
Cancer, *n* (%)	18 (11.8)	13 (8.7)
**Clinical features**		
MADRS baseline score, mean (SD)	29.7 (6.1)	29.8 (6.6)
Years from depression diagnosis, mean (SD)	11.0 (14.4)	10.2 (14.9)
History of suicide attempts, *n* (%)	5 (3.3)	4 (2.7)
PDD/DDD at baseline, mean (SD)	0.84 (0.43)	1.04 (0.28)
Titration days, mean (SD)	7.4 (6.2)	7.7 (5.4)

*SSRI* selective serotonin reuptake inhibitor, *MADRS* Montgomery–Åsberg Depression Rating Scale, *n* number of study participants with the index variable, *PDD/DDD* ratio between prescribed and defined daily doses, *SD* standard deviation

^a^11 missing data

^b^Versus single/widow; 7 missing data

### Study Outcomes

There were no differences between vortioxetine and SSRI groups in terms of MADRS improvement, as estimated by mean change scores across follow-up visits (−8.9 ± 9.5 versus −9.8 ± 10.5, *p* = 0.67 at T1; −11.0 ± 9.9 versus −13.6 ± 12.1, *p* = 0.08 at T2; −11.8 ± 10.6 versus −14.0 ± 11.6, *p* = 0.12 at T3).

**Fig. 2:**
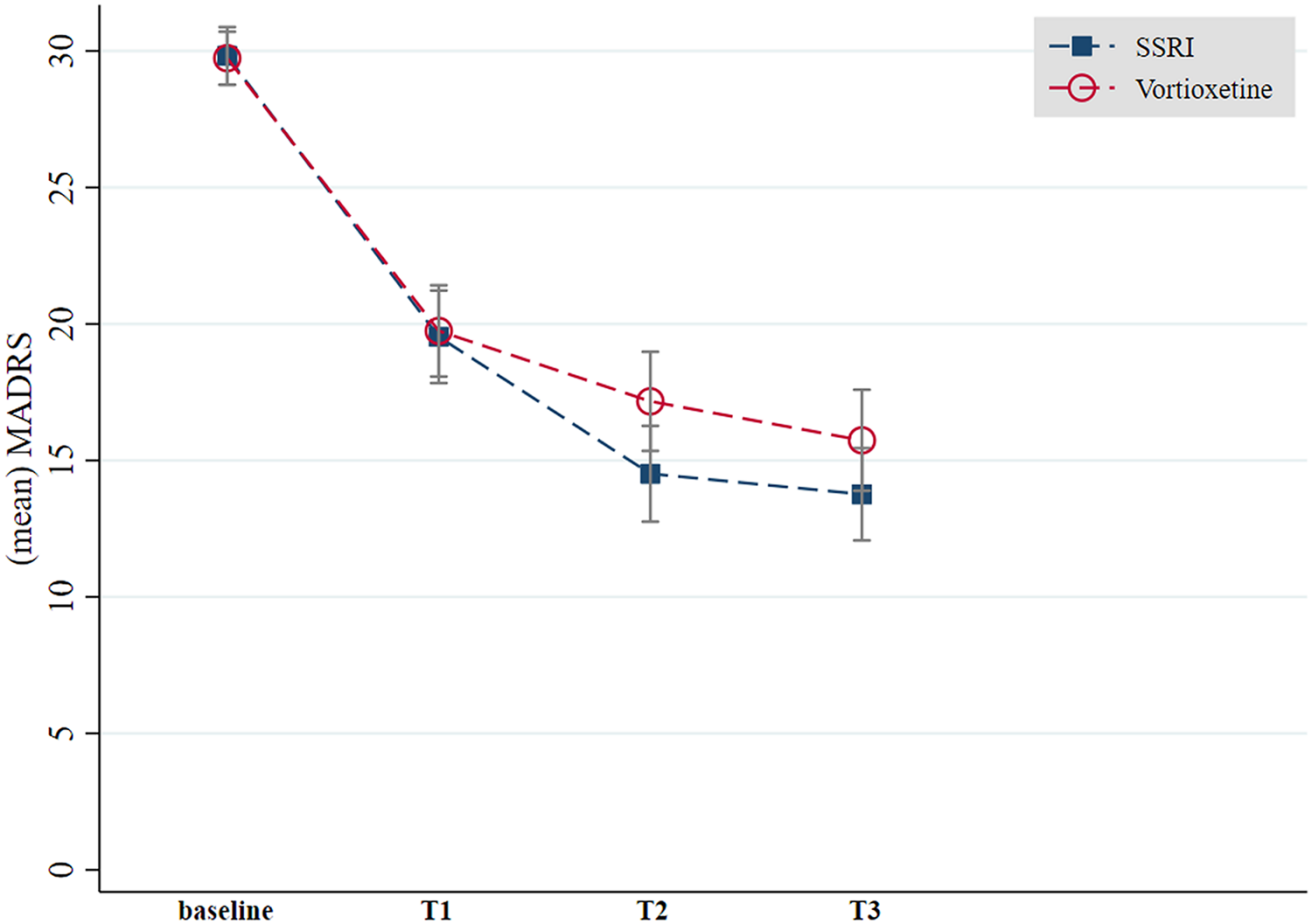
MADRS mean scores over time. *MADRS* Montgomery–Åsberg Depression Rating Scale, *SSRI* selective serotonin reuptake inhibitor group. Mean MADRS scores with relevant standard errors at different timepoints in vortioxetine and SSRI groups are shown

Handling missing data, a repeated measure mixed effect model confirmed these results (Table 2). A reduction of MADRS scores was observed over time among both groups. However, there were no statistically significant differences between vortioxetine and SSRI treatments. Figure 2 shows MADRS mean scores over time by vortioxetine and SSRI groups.

**Table 2:** Repeated measure mixed effects models

	Model 1			Model 2		
	Coefficient (95% CI)	Standard error	*p*-value	Coefficient (95% CI)	Standard error	*p*-value
Treatment^a^	1.07 (−0.57; 2.71)	0.84	0.200	−0.75 (−3.21; 1.70)	1.25	0.548
Time	−4.94 (−5.33; −4.55)	0.20	< 0.001	−5.32 (−5.87; −4.77)	0.28	< 0.001
Treatment × time	–	–	–	0.78 (− 0.003; 1.57)	0.40	0.051

*CI* confidence interval

^a^Vortioxetine versus SSRIs (selective serotonin reuptake inhibitors)

**Fig. 3:**
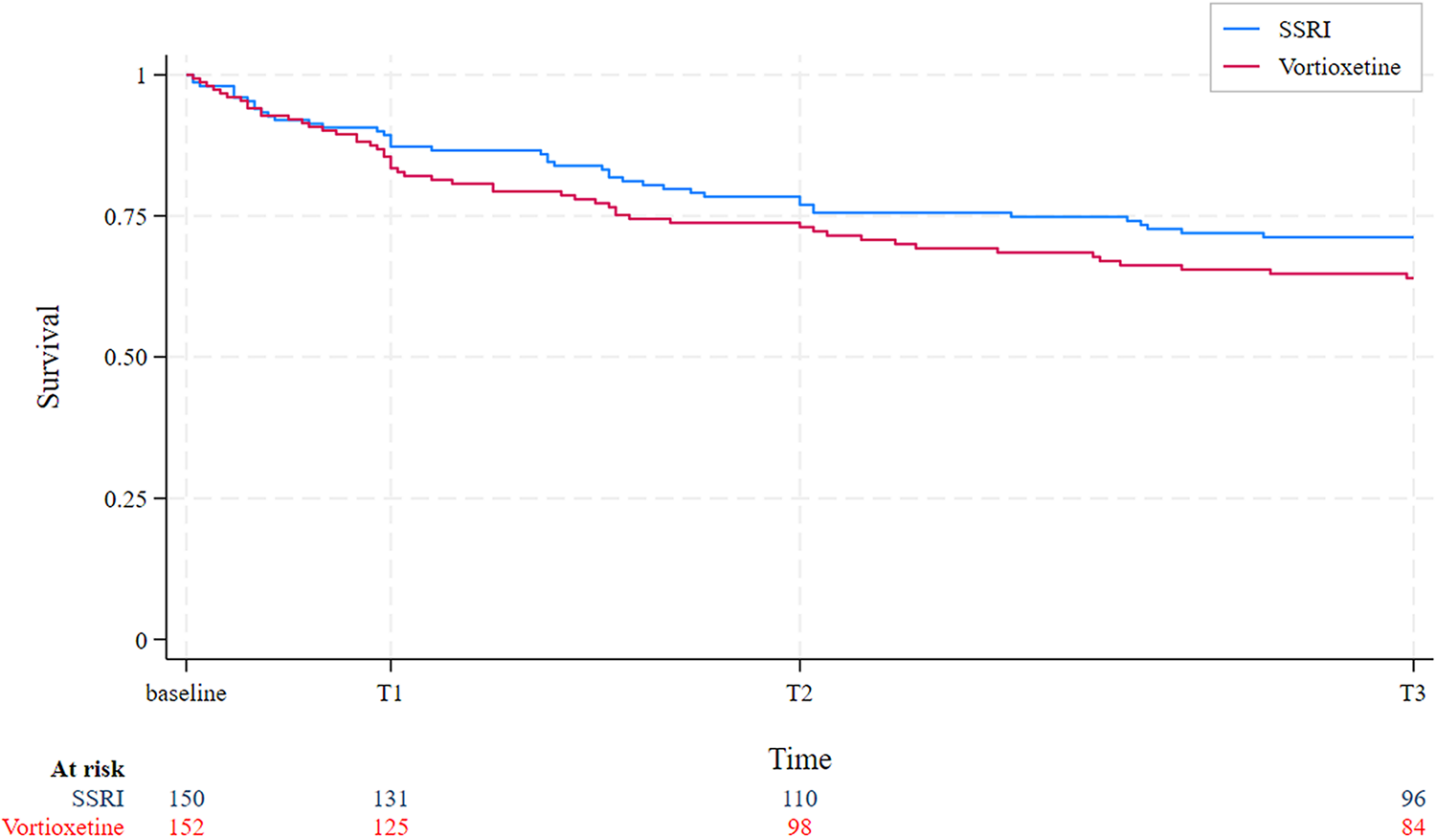
Kaplan–Meier survival estimates (failure discontinuation). *SSRI* selective serotonin reuptake inhibitor group

In addition, baseline PDD/DDD did not influence MADRS improvement at the endpoint (regression coefficient: −0.52, *p* = 0.76). The subgroup analysis, including only individuals who did not discontinue the allocated drug (*n* = 180), confirmed that there was no difference (*p* = 0.50) in MADRS mean change scores at the endpoint (T3) between vortioxetine (*n* = 84; −16.9 ± 9.1) and SSRIs (*n* = 96; −17.7 ± 10.3). Regarding secondary outcomes, no statistically significant differences in either response (44.1% in the vortioxetine group versus 53.3% in the SSRI group; *p* = 0.11) or remission (25.7% in the vortioxetine group versus 34.7% in the SSRI group; *p* = 0.09) rates were estimated. Again, subgroup analyses including only drug continuers (*n* = 180) further confirmed these findings for both clinical response (*p* = 0.98) and remission (*p* = 0.36). Moreover, vortioxetine was not better than SSRI groups regarding discontinuation owing to any cause (38.0 versus 30.2%; *p* = 0.17). Consistently, no differences in discontinuation due to either side effects (20.4 versus 15.1%; *p* = 0.25) or inefficacy (10.9 versus 8.6%; *p* = 0.52) were detected. Survival analyses corroborated these findings, showing no statistically significant differences in time to discontinuation between vortioxetine and SSRI groups (HR = 1.31; 95% CI = 0.87 to 1.97; *p* = 0.19), even considering death as a competing risk (survival function: Fig. 3; cumulative incidence estimate: Supplementary Fig. 1). Regression models showed that baseline PDD/DDD did not influence also secondary outcomes at the endpoint, i.e. response (coefficient: −0.17; *p* = 0.58), remission (coefficient: 0.06, *p* = 0.85), and discontinuation (coefficient: 0.31, *p* = 0.36). A comparative summary of results for secondary outcomes is presented in Table 3.

**Table 3:** Secondary outcomes: summary of findings

	Vortioxetine group (*n* = 152)	SSRI group (*n* = 150)	*p*-value
Response, *n* (%)			
Overall sample	67 (44.1)	80 (53.3)	0.11
Subgroup of drug continuers only (*n* = 180)	57 (67.9)	65 (67.7)	0.98
Remission, *n* (%)			
Overall sample	39 (25.7)	52 (34.7)	0.09
Subgroup of drug continuers only (*n* = 180)	32 (38.1)	43 (44.8)	0.36
Discontinuation, *n* (%)			
Any cause discontinuation	52 (38.0)	42 (30.2)	0.17
Discontinuation due to side effects	28 (20.4)	21 (15.1)	0.25
Discontinuation due to inefficacy	15 (10.9)	12 (8.6)	0.52

*SSRI* selective serotonin reuptake inhibitor, *n* number of study participants with the index variable

## Discussion

### Summary and Interpretation of Findings

The VESPA study [11, 17] stands as the largest clinical trial to date to primarily compare vortioxetine with SSRIs in treating late-life depression. Through the present post-hoc analysis, we investigated whether vortioxetine was superior to SSRIs regarding efficacy and tolerability in a subsample of older people with moderate-to-severe depression (MADRS score ≥ 20). Our primary outcome, based on mean changes in MADRS scores, revealed that vortioxetine was not superior to SSRIs over a 6-month treatment period. This finding was confirmed after excluding study participants who discontinued treatment or had missing information on discontinuation data. However, SSRIs demonstrated slightly better, albeit not statistically significant, response and remission rates.

Moreover, exploring specific reasons for discontinuation, such as side effects or inefficacy, there were no significant differences between the two treatment arms. These findings are consistent with established evidence in this field in younger target populations. Indeed, a relatively recent systematic review and meta-analysis, including 20 trials and 8547 participants, indicated that vortioxetine outperformed placebo, but not SSRIs, in terms of response to treatment, remission, and drop out due to adverse events [23]. Nonetheless, it is worth mentioning that the specific design of our study may account for possible intrinsic disadvantages for the group allocated to vortioxetine. First, while just one treatment option was allowed for people randomized to vortioxetine, the specific agent for the group randomized to SSRIs was chosen according to clinical judgment. This wider range of possible psychopharmacological options may have represented a significant clinical advantage for the SSRI arm, as it allowed clinicians to personalize treatment: for instance, clinicians might have chosen sertraline to avoid drug interactions [24], paroxetine for its anxiolytic properties [25] or fluoxetine for a more activating effect [26]. Second, even though flexible drug dosing allowed the trial to replicate real-world clinical practice more closely, this might have also generated some discrepancies between treatment arms. Indeed, we observed that vortioxetine was prescribed at lower doses than SSRIs, with a mean daily dose corresponding to around 80% of PDD/DDD. This is certainly consistent with common clinical practice, in which vortioxetine is often prescribed at low doses (e.g. 5 mg) in older people and titrated up to a maximum of 10 mg, in line with EMA summary of drug characteristics and recommendations [27]. This is a key point since the effectiveness of vortioxetine seems to follow a dose–response relationship with 20 mg (PDD/DDD = 2.0) being significantly more effective than 10 mg (PDD/DDD = 1.0) [28]. Post-hoc analyses from 12 short-term, fixed-dose, randomized, placebo-controlled trials in people older than 55 years of age pointed out that vortioxetine outperformed placebo in terms of response rates at 10 and 20 mg daily, while remission rates were significantly higher only at 20 mg daily [9]. However, while we cannot rule out the hypothesis that higher doses—at least in the vortioxetine group—might have led to somewhat improved clinical outcomes, we should consider that the use of lower doses may help minimize adverse reactions. Indeed, some study participants may not have reached the target dose due to side effects, consistently with the primary analysis showing that increased discontinuation rates due to adverse events in those treated with vortioxetine [11]. In addition, the greater familiarity of prescribers with SSRIs compared to vortioxetine may have affected the dose titration of selected drugs in the absence of blinding.

### Study Limitations

Some limitations should be acknowledged. First, even though post-hoc analyses may be useful to provide a means by which novel hypotheses can be formally assessed, intrinsic issues related to their design need to be considered [29]. In particular, it should be noted that the primary study was designed as a superiority trial, while the comparison of efficacy between vortioxetine and SSRIs might be better suited to a non-inferiority or equivalent study design. Thus, the exploratory nature of this secondary analysis should be considered when interpreting results. Second, the lack of blinding may have introduced possible sources of performance and detection bias. Specifically, after randomization and throughout the trial, only outcome assessors were blinded, while both clinicians and study participants were aware of treatment allocation. In addition, the blinding of biostatisticians, as implemented in the primary study [11], could not be guaranteed by this post-hoc analysis. Another limitation is that add-on treatments with drugs such as second-generation antipsychotics and lithium were forbidden at baseline but then allowed during the follow-up. Even though low rates of additional treatments were found in our primary analysis [11], we cannot rule out that the combination of these agents with antidepressants might have—at least partially—influenced the study outcomes. In addition, while we followed a pragmatic approach in selecting all SSRIs as comparators, it should be acknowledged that, for the specific purpose of this post-hoc analysis, a head-to-head comparison with a single SSRI agent would have been more appropriate. Finally, it should be noted that major depressive disorders represent heterogeneous conditions that requires careful characterization and clustering, according to specific clinical features [30, 31]. Nonetheless, in this study we did not collect information on relevant clinical specifiers that may influence treatment outcomes, such as anxious distress [32], atypical symptoms [33], mixed features [34] and psychotic features [35]. This is an important point considering that, for example, vortioxetine has shown to be particularly effective in people with high levels of anxiety symptoms associated with depression [36, 37]. Clinical trials targeting depression subtypes in older people should be one of the main aims of future research in this field [38, 39].

## Conclusions

This post-hoc, exploratory analysis did not highlight any advantage of vortioxetine over SSRIs in older adults with moderate-to-severe depression. No differences were found in terms of depressive symptom improvement, clinical response, remission and discontinuation rates. Additional trials using fixed and targeted doses of vortioxetine are needed to understand its role of in the treatment of late-life depression. Such studies may benefit from a more thorough evaluation of clinical specifiers of major depressive episodes, on which vortioxetine and SSRIs may have differential effectiveness.

## Supplementary Information

Below is the link to the electronic supplementary material.

Supplementary file1 (PDF 212 KB)

## Declarations

### Availability of data and material

The dataset will be made available upon motivated request.
