# Supplementary material for: Efficacy and Tolerability of Vortioxetine Versus Selective Serotonin Reuptake Inhibitors for Late-Life Depression: A Post-hoc Analysis of the VESPA Study
Source: Drugs Aging. 2025 Jul 18;42(8):771–80. doi: 10.1007/s40266-025-01231-3 (PMC12313815; doi:10.1007/s40266-025-01231-3)
Supplement: Supplementary file 1 — Supplementary file1 (PDF 212 KB) [file 40266_2025_1231_MOESM1_ESM.pdf]

Efficacy and tolerability of vortioxetine vs. selective serotonin reuptake inhibitors for late-life depression: A post-hoc analysis of the VESPA study

Drugs & Aging

Francesco Bartoli [1], Daniele Cavaleri [1], Ilaria Riboldi [1,*], Tommaso Callovini [1], Cristina Crocamo [1], Chiara Gastaldon [2], Andrea Aguglia [3,4], Camilla Callegari [5], Simone Cavallotti [6], Stefania Chiappini [7,8], Marco Cruciata [9], Armando D'Agostino [6,10], Irene Espa [3], Luigi Grassi [9], Marta Ielmini [5], Silvia Mammarella [11], Giovanni Martinotti [7], Marianna Rania [12], Alessandro Rodolico [13,14], Rita Roncone [11], Valentina Roselli [15], Cristina Segura-Garcia [12,16], Maria Salvina Signorelli [13,17], Lorenzo Tarsitani [15], The VESPA Study Group [§], Giovanni Ostuzzi [2,○], Giuseppe Carrà [1,○]

[1] School of Medicine and Surgery, University of Milano-Bicocca, Monza, Italy.
[2] World Health Organization Collaborating Centre for Research and Training in Mental Health and Service Evaluation, Department of Neuroscience, Biomedicine and Movement Sciences, Section of Psychiatry, University of Verona, Verona, Italy.
[3] Department of Neuroscience, Rehabilitation, Ophthalmology, Genetics, Maternal and Child Health, Section of Psychiatry, University of Genoa, Italy.
[4] IRCCS Ospedale Policlinico San Martino, Genoa, Italy.
[5] Department of Medicine and Surgery, Section of Psychiatry, University of Insubria, Varese, Italy.
[6] Department of Mental Health and Addiction, ASST Santi Paolo e Carlo, Milan, Italy.
[7] Department of Neurosciences, Imaging and Clinical Sciences, University "G. D'Annunzio", Chieti, Italy.
[8] UniCamillus International Medical Sciences University, Rome, Italy.
[9] Institute of Psychiatry, Department of Neuroscience and Rehabilitation, University of Ferrara, Ferrara, Italy.
[10] Department of Health Sciences, University of Milan, Milan, Italy.
[11] Department of Life, Health and Environmental Sciences, University of L'Aquila, L'Aquila, Italy.
[12] Outpatient Unit for Clinical Research and Treatment of Eating Disorders, University Hospital Renato Dulbecco, Catanzaro, Italy.
[13] Department of Clinical and Experimental Medicine, Institute of Psychiatry, University of Catania, Catania, Italy.
[14] Technical University of Munich, TUM School of Medicine and Health, Department of Psychiatry and Psychotherapy, Klinikum rechts der Isar, Munich, Germany
[15] Department of Human Neuroscience, Sapienza University of Rome, Rome, Italy.
[16] Psychiatry Unit, Department of Medical and Surgical Sciences, University Magna Graecia of Catanzaro, Catanzaro, Italy.
[17] Oasi Research Institute - IRCCS, Troina, Italy.

[§] The VESPA Study Group includes the following investigators:
(A) University of Verona: Corrado Barbui, Marco Cesca, Chiara Gastaldon, Alessia Metelli, Giulia Michencig, Michela Nosé, Filippo Ogheri, Giovanni Ostuzzi, Davide Papola, Marianna Purgato, Federica Robbi, Enrico Sterzi, Beatrice Todesco, Giulia Turrini, Elisa Zanini;
(B) University of Catania: Eugenio Aguglia, Andrea Birgillito, Emanuele Bisso, Gabriele Avincola, Salvatore Canonico, Cecilia Chiarenza, Sara Coloccini, Leonardo Marano, Alessandro Moncada, Alessandro Rodolico, Maria Salvina Signorelli, Serena Sturiale, Luca Zambuto;
(C) University of Chieti-Pescara: Maria Chiara Alessi, Clara Cavallotto, Chiara Di Natale, Andrea Miuli, Alessio Mosca, Mauro Pettorusso, Antonella Sociali, Antonio Tambelli;

(D) University Magna Graecia of Catanzaro: Elvira Anna Carbone, Pasquale De Fazio, Renato de Filippis, Ettore D'Onofrio, Valentina Pugliese, Marianna Rania, Cristina Segura-Garcia;
(E) University of Ferrara: Doriana Carosielli, Marco Cruciata, Luigi Grassi, Barbara Ronchi, Federico Marconi, Gabriele Simonelli, Marco Marella, Marta Gancitano, Samantha Romito, Chiara Zannini, Luigi Zerbinati;
(F) University of Genoa: Andrea Aguglia, Irene Espa, Matteo Gari, Alice Trabucco, Veronica Villa;
(G) University of L'Aquila: Silvia Mammarella, Rita Roncone, Sasha Del Vecchio, Laura Giusti;
(H) University of Milano Statale: Stefano Bonora, Simone Cavallotti, Armando D'Agostino, Manuela De Palma, Giulia Fior, Barbara Giordano, Gianmarco Ingrosso, Margherita Oresti, Federico Wiedenmann;
(I) University of Milano-Bicocca: Francesco Bartoli, Giuseppe Carrà, Tommaso Callovini, Daniele Cavaleri, Bianca Bachi, Angela Calabrese, Riccardo Matteo Cioni, Federico Moretti, Aurelia Canestro, Chiara Alessandra Capogrosso, Pierluca Guzzi, Marco Morreale, Christian Nasti, Dario Palpella, Susanna Piacenti, Pietro Morello, Gianna Bernasconi, Claudia Cumerlato, Annamaria Lax, Alessandra Ornaghi;
(J) University La Sapienza (Rome): Bianca Della Rocca, Liliana Todini, Corinna Pancheri, Silvia Passeri, Irene Pinucci, Liliana Todini;
(K) University of Insubria (Varese): Camilla Callegari, Ivano Caselli, Marta Ielmini, Celeste Isella;
(L) IRCCS Istituto di Ricerche Farmacologiche Mario Negri: Angelo Barbato, Massimo Cartabia, Barbara D'Avanzo, Igor Monti, Mauro Tettamanti.

* Corresponding author:
Ilaria Riboldi, School of Medicine and Surgery, University of Milano-Bicocca, via Cadore 48, 20900 Monza, Italy.
ilaria.riboldi@unimib.it
° *Shared last authors*.

Supplementary Figure 1. Competing-risks regression (cumulative incidence).

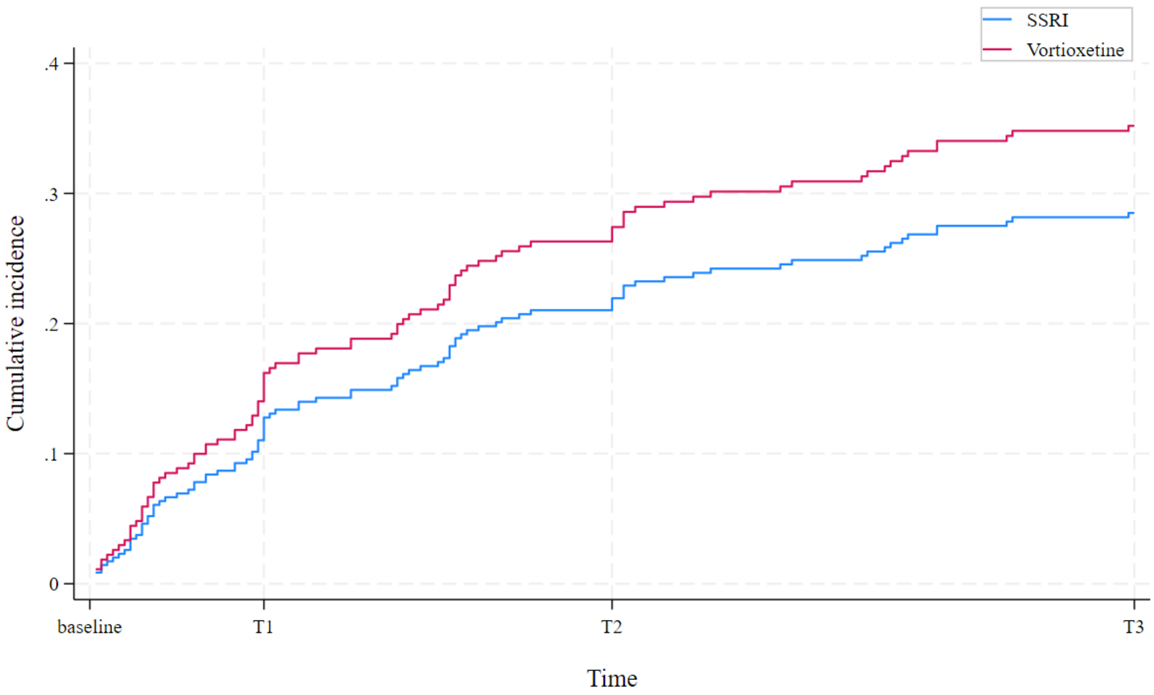

SSRI = selective serotonin reuptake inhibitors.
